# Supplementary material for: Structural basis for the activation of PLC-γ isozymes by phosphorylation and cancer-associated mutations
Source: eLife. 2019 Dec 31;8:e51700. doi: 10.7554/eLife.51700 (PMC7004563; doi:10.7554/eLife.51700)
Supplement: Supplementary file 1. [file elife-51700-supp1.docx]

**Supplementary file 1.** Crystallization statistics for PLC-γ1.

|  | **native** | **Gd^3+^-derivative*^a^*** |
| --- | --- | --- |
| **Data collection** |  |  |
| wavelength (Å) | 1.0000 | 1.7115 |
| space group | P2_1_2_1_2_1_ | P2_1_2_1_2_1_ |
| unit cell dimensions |  |  |
| a, b, c (Å) | 70.8, 82.4, 228.3 | 71.1, 83.4, 232.3 |
| α, β, γ (deg.) | 90.0, 90.0, 90.0 | 90.0, 90.0, 90.0 |
| resolution (Å) | 41.23-2.46 (2.50-2.46) | 49.04-2.98 (3.04-2.98) |
| unique reflections | 49,443 (2,380) | 51,779 (2,158) |
| R_merge_***^b^*** | 12.3 (69.2) | 9.9 (78.8) |
| <I/σ>***^c^*** | 16.9 (3.3) | 12.6 (1.3) |
| completeness (%) | 99.9 (97.2) | 95.6 (80.3) |
| redundancy | 8.1 (6.8) | 4.3 (3.3) |
| CC_1/2_ | 99.7 (79.5) | 99.6 (59.7) |
|  |  |  |
| **Refinement*^e^*** |  |  |
| resolution (Å) | 41.23-2.46 |  |
| no. of reflections  (working set/test set) | 45,911/3,526 |  |
| R/R_free_***^d^*** | 20.2/24.5 |  |
| no. non-hydrogen atoms |  |  |
| protein | 8,598 |  |
| water | 246 |  |
| Ca^2+^ ion | 1 |  |
| Na^+^ ion | 1 |  |
| RMSD |  |  |
| bond lengths (Å) | 0.003 |  |
| bond angles (deg.) | 0.600 |  |
| average B factor (Å^2^) |  |  |
| protein | 37.8 |  |
| water | 32.3 |  |
| Ca^2+^ ion | 42.4 |  |
| Na^+^ ion | 56.6 |  |
| Ramachandran plot (%) |  |  |
| favored | 96.5 |  |
| allowed | 3.5 |  |
| disallowed | 0 |  |

Numbers in parentheses refer to data for the highest-resolution shell. Each dataset was collected from a single crystal.

***a***: Merging statistics were calculated with Friedel mates treated separately.

***b***: R_merge_ = 100Σ|I-<I>|/ΣI, where *I* is the integrated intensity of a measured reflection.

***c***: <I/σ> is the mean signal-to-noise ratio, where *I* is the integrated intensity of a measured reflection and σ is the estimated error in the measurement.

***d***: R = 100Σ|F_o_-F_c_|/ΣF_o_, where *F_o_* and *F_c_* are the observed and calculated structure factor amplitudes, respectively. R_free_ is calculated as R using 7% of the total reflections that were randomly excluded from refinement.

***e***: Refinement was performed with riding hydrogens.
